# Identifying a Novel Bile Salt Hydrolase from the Keystone Gut Bacterium *Christensenella minuta*

**DOI:** 10.3390/microorganisms9061252

**Published:** 2021-06-09

**Authors:** Guillaume Déjean, Héloïse Tudela, Lisa Bruno, Déborah Kissi, Georges Rawadi, Sandrine P. Claus

**Affiliations:** 1 Ysopia Bioscience, 17 Place de la Bourse, 33076 Bordeaux, France; guillaume.dejean@ysopia.bio (G.D.); heloise.tudela@ysopia.bio (H.T.); lisa.bruno@ysopia.bio (L.B.); deborah.kissi@ysopia.bio (D.K.); georges.rawadi@ysopia.bio (G.R.); 2 ImmunoConcEpT, 146 rue Léo Saignat, 33076 Bordeaux, France

**Keywords:** gut microbiome, bile acids, bile salt hydrolase

## Abstract

*Christensenella minuta* are human gut dwelling bacteria that have been proposed as key members of the gut microbiome, regulating energy balance and adiposity of their host. We formerly identified that a novel strain of *C. minuta* (strain DSM33407) boosted microbiota diversity and stimulated deconjugation of the primary bile acid taurocholic acid in human samples. However, there is no description of a bile salt hydrolase (BSH) protein carried in the genome of *C. minuta*. Here, we identified and cloned a protein from *C. minuta’s* genome that carries a potent BSH activity, which preferentially deconjugates glycine-conjugated bile acids. We then retrieved 14,319 putative BSH sequences from the NCBI database and filtered them using the UHGP database to collect a total of 6701 sequences that were used to build the most comprehensive phylogenetic tree of BSH-related enzymes identified in the human microbiome so far. This phylogenetic tree revealed that *C. minuta’s* BSH amino acid sequence clusters away from others with a threshold of 70% identity. This is therefore the first description of *C. minuta’s* BSH protein, which may be involved in its unique role within the human gut microbial ecosystem.

## 1. Introduction

*Christensenella minuta* is a low abundance Gram-negative human gut commensal bacterial species of the Firmicutes phylum that has been suggested as a key modulator of adiposity [1,2]. In addition, it has been reported to consistently decrease in several human obese cohorts, suggesting that it carries a unique function associated with the regulation of energy metabolism [1,3,4,5,6]. To date, only one strain of *C. minuta* (DSM 22607, referred to further in the text as *Cm*DSM22607) is available in culture collections, which is the original type strain described by Morotomi et al. in 2012 [7]. In a recently published study, we demonstrated the anti-obesity potential of the novel strain *Christensenella minuta* DSM 33407 (*Cm*DSM33407) in a diet-induced obesity (DIO) mouse model [8]. In this article, we also evaluated the effects of this new strain on the gut microbiome of obese donors using a SHIME® model and demonstrated that it promotes gut bacterial diversity, restoring the altered Bacteroides/Firmicutes ratio typical of a dysbiotic obese microbiota [9]. This effect was associated with a stimulation of the production of short chain fatty acids (SCFAs) and tended to increase the cholic acid/taurocholic acid (CA/TCA) ratio in the distal part of the colon [8], indicating that the function of the microbiota supplemented with *C. minuta* DSM 33407 was shifted towards a higher saccharolytic metabolism associated with more efficient deconjugation of primary bile acids.

Bile acid metabolism has long been known to be regulated by gut microbes since they facilitate their recycling through the enterohepatic cycle, which contributes to cholesterol homeostasis [10]. In the upper gastrointestinal tract, bile acids regulate lipid absorption, which is an important aspect of the regulation of energy balance [11,12]. Beyond this well-known function, bile acids are also key mediators of the gut microbiome–host metabolism crosstalk since they act as signaling molecules, triggering host responses via the metabolic pathways controlled by the Farnesoid X Receptor (FXR) and the Takeda G protein Receptor 5 (TGR-5) [10]. Hence, they have been suggested as gut microbiota-derived hormones that regulate several systemic functions involved in energy, immune and even brain metabolisms [13,14].

Bile acid deconjugation occurs in the gut through the bacterial enzyme Bile Salt Hydrolase (BSH; EC 3.5.1.24), a choloylglycine hydrolase enzyme that catalyzes the hydrolysis of the amino acid side chain of conjugated bile acids [11]. This action facilitates the reabsorption of unconjugated bile acids by passive diffusion and allows further metabolism by bacteria to produce secondary bile acids (e.g., deoxycholic and lithocholic acid). Importantly, BSH enzymes are structurally close to penicillin acylases (EC:3.5.1.11) and acid ceramidases (EC:3.5.1.23), which makes them difficult to identify using high throughput bioinformatic methods that use automatic annotations.

BSH activity is a conserved microbial adaptation that is unique to gut-associated bacteria and is distributed across the major bacterial divisions and archaeal species in the gastrointestinal tract. Indeed, BSH has been identified in several well-studied gut microbes such as *Bifidobacterium* spp. [15,16], *Lactobacillus* spp. [17,18], *Enterococcus* spp. [19] and *Methanobrevibacter* spp. [20]. Although most BSHs have been studied in Gram-positive organisms, the BSHs expressed by Gram-negative *Bacteroides* spp. were recently investigated to reveal interesting selectivity patterns [21]. Bioinformatic analyses have shown that BSH enzymes are widely encoded in the human gut microbiome across all ethnicities [22]. It has been proposed that one of the reasons for the conservation of this enzyme in many members of the gut microbiota is its contribution to resistance to bile acid toxicity, conferring an evolutionary advantage to its carriers [23]. Yet, this vision has been challenged [24] and a recent study indicates that BSH enzymes modulate the bacteria’s transcriptome in response to bile acids, hence adapting their fitness to the environment [17]. Interestingly, the type species *C. minuta* DSM22607 (*Cm*DSM22607) has been described as highly tolerant to bile acids [7], suggesting that it possesses a molecular engine highly efficient in detoxifying bile acids. Hence, we hypothesized that *Cm*DSM33407 carries a functional BSH. Here, we describe, for the first time, the specific BSH of *Cm*DSM33407.

## 2. Materials and Methods

### 2.1. Bacterial Growth and Bile Acid Tolerance Assay

*Christensenella minuta* DSM 22607 (NCBI accession no. NZ_CP029256.1) and DSM 33407 were grown on pre-reduced Gifu anaerobic modified medium (GAMm, Hyserve, Uffing, Germany) during 3 days at 37 °C under anaerobic conditions (Coy anaerobic chamber; H_2_ 5%, CO_2_ 5%, N_2_ 90%). All bile acid substrates (i.e., OxGall, GCA, GCDCA, TCA and TCDCA) were purchased from Sigma-Aldrich (Merck, Darmstadt, Germany).

The ability of *C. minuta* DSM22607 and DSM33407 to grow in the presence of bile acids was evaluated in 10 mL of modified GAM media broth (Hyserve, Uffing, Germany) supplemented with 0%, 2%, 4%, 6%, 8% *w*/*v* OxGall (Difco Laboratories supplied by BD Diagnostics, Le Pont de Claix, France; equivalent to 0%, 20%, 40%, 60% and 80% bile, as per the manufacturer’s description). Each condition was inoculated at 1% *v*/*v* with a preculture of the *C. minuta* strains harvested after 72 h of growth. The bacterial culture was then incubated for 96 h at 37 °C under anaerobic conditions. The assay was performed in duplicates. The growth culture was evaluated by measuring optical density (OD 600 nm) in cuvettes using a Biowave Cell Density Meter CO8000 (Biochrom Ltd., Cambridge, UK) at 0 h, 24 h, 48 h, 72 h and 96 h. To determine the bacteria viability for each condition, CFU counting was performed at 48 h and 96 h in modified GAM agar plates and was incubated at 37 °C for 5 days under anaerobic conditions.

### 2.2. Cloning of C. minuta DSM 33407 BSH Gene

PCR amplified fragments of the full-length ORF of the BSH gene of *C. minuta* DSM33407 were obtained using the Q5 high fidelity polymerase (New England Biolabs, Evry, France) with the primers *Cm33407BSH*-F (5′-TACTTCCAATCCAATGCCATGTGTACAGCAATAACGTATTATACAAA-3′) and *Cm33407BSH*-R (5′-TTATCCACTTCCAATGTTATTAGTAATTCCGATAGTTAATCTGCTG-3′). PCR products that contained appropriate pMCSG complementary sequences for subsequent ligation were cloned into pMCSG53 plasmid providing an N-terminal His_6_-tag [25,26]. Successful cloning was confirmed by amplifying the target gene fragment (978 bp) from selected colonies (Taq’Ozyme polymerase from Ozyme, Paris, France) and sent for DNA sequencing (Genewiz, Leipzig, Germany).

### 2.3. Expression and of Recombinant BSH

Recombinant proteins were produced in *E. coli* BL21 (DE3) cells cultured in 500 mL of LB broth containing ampicillin (50 μg·mL^−1^) at 37 °C (200 rpm). Cells were grown to mid-exponential phase (OD_600_ ~ 0.4 to 0.6). Overexpression was induced by adding isopropyl *β*-D-thiogalactopyranoside (IPTG) to a final concentration of 0.5 mM and the cultures were further grown at 16 °C (200 rpm) for 18 h. The cells were harvested by centrifugation (7000× *g* for 20 min at 4 °C), sonicated and His_6_-tagged recombinant proteins were purified via immobilized nickel affinity chromatography (His-Trap; Cytiva, Marlborough, MA, USA) utilizing a one-step gradient elution up to 100% elution buffer containing 20 mM sodium phosphate, pH 7.4, 500 mM NaCl and 500 mM imidazole using an Akta Pure protein purification FPLC system (Cytiva, Marlborough, MA, USA). The purity of the recombinant proteins was determined by SDS/PAGE and the final concentration was determined from calculated molar extinction coefficients at 280 nm using a Nanodrop One (Thermofisher Scientific, Illkirch, France).

### 2.4. Enzymatic Assays

A fluorometric glycine assay kit (ab211100, abcam, Paris, France) and a colorimetric taurine assay kit (Merck, Darmstadt, Germany) were used to respectively determine the glycine and taurine amounts released by the action of the raw bacteria lysates in the preliminary bile acid deconjugation assay. The activity of the purified recombinant BSH was determined by measuring the amount of the amino acids released from conjugated bile salts using the ninhydrin assay as described by Tanaka and coworkers [27] Briefly, to 180 μL of reaction buffer (0.1 M citrate buffer, pH 6.0), 10 μL of purified *Cm*33407BSH, 10 μL of 100 mM of bile acids (either GCA: glycocholic acid, GCDCA: glycochenodeoxycholic acid, TCA: taurocholic acid, TCDCA: taurochenodeoxycholic acid) and 10 mM dithiothreitol (DTT) were added. The reaction was carried out at 37 °C. A control reaction without the enzyme and one with the denatured enzyme (10 min, 90 °C) were also performed. A 50 μL sample was taken after a given time of reaction and was mixed immediately with 50 μL of 15% (*w*/*v*) trichloroacetic acid. This sample was centrifuged at 16,000× *g* for 2 min to remove the precipitate. A 50 μL aliquot of this first reaction was then mixed with 950 μL of ninhydrin reagent (0.25 mL of 1% (*w*/*v*) ninhydrin in 0.5 M citrate buffer pH 5.5; 0.6 mL of glycerol; and 0.1 mL of 0.5 M sodium-citrate buffer pH 5.5). The mixture was boiled for 13 min and chilled on ice for 3 min. The absorbance at 570 nm was measured.

### 2.5. Protein Sequence and In Silico Structural Analysis

The full-length protein *Cm*33407BSH was screened for the presence of a putative signal peptide sequence using SignalP (version 5.0), LipoP 1.0 and PSORTb [28,29,30]. Alignments with representative BSH sequences were achieved using Clustal Omega [31] and ESPript 3 software [32]. In ESPript 3.0, strict β-turns are indicated as TT letters, strict α-turns as TTT and the η symbol refers to a 310-helix. The 3D model of *Cm*33407BSH was achieved using advanced remote homology detection methods via the Phyre2 web portal [33]. The structure representations were made using the PyMOL Molecular Graphics System (v2.4.0, by Schrödinger).

### 2.6. BSH Alignment, Phylogenetic Analysis and Clustering

The BSH protein sequence from *C. minuta* DSM 22607 was retrieved from the NCBI protein database (GenBank: AYH40638.1). The amino acid sequences of the BSH from *C. minuta* DSM 22607 and DSM 33407 were aligned using Muscle (v.3.8.31) with default parameters and the alignment was viewed using MView [34,35].

A dataset of 6701 putative BSH sequences from the human gut was constructed using the flowchart shown in Figure S1. Briefly, a total of 14,319 sequences were extracted from the NCBI refseq protein database based on their annotation, length and sources. The sequences were blasted (Blastp v2.9.0+) against the Unified Human Gastrointestinal Protein (UHGP-100 v1.0, downloaded on 2 December 2020) catalog and the gastrointestinal tract protein database from the Human Microbiome Project (HMP) [36,37,38,39] Out of the 14,319 putative BSH sequences from the NCBI refseq protein database, the sequences sharing 85% identity or more with at least one sequence of the UHGP database were selected to form a dataset of 6701 putative BSH sequences from the human gut. A clustering analysis was performed using CD-hit (v.4.6.1) on this dataset using a 70% identity threshold and a word size of 4.

For each tree, the selected sequences were aligned using Muscle (v.3.8.31) with default parameters [34]. Gblocks (v0.91b) with default parameters was used to eliminate divergent and poorly aligned sequences in the alignment [40]. MEGA-X for macOS (v10.1.8) was used to create the phylogenetic tree [41,42]. The initial tree was obtained using neighbor joining and checked using the maximum likelihood method, using 1000 iterations to validate the bootstrap values.

## 3. Results

### 3.1. Christensenella minuta DSM 33407 Displays High Resistance to Bile Acids

**Figure 1:**
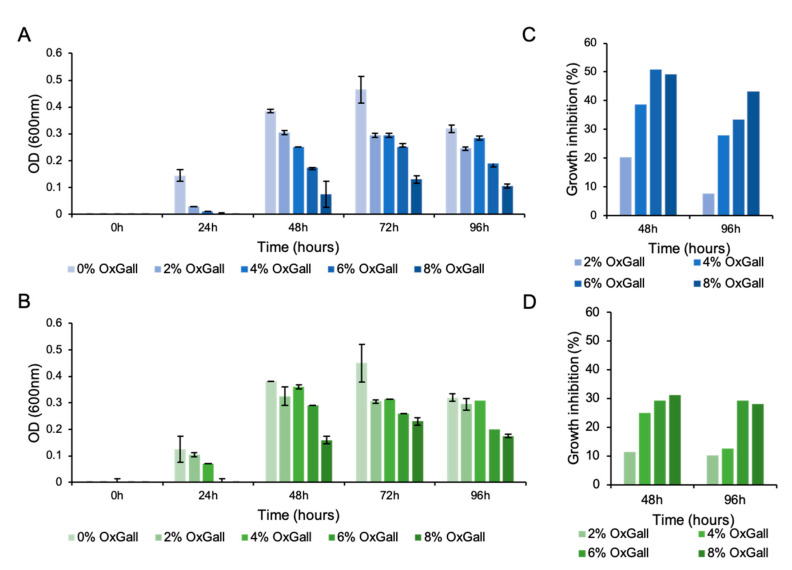
Bile acid resistance by *Christensenella minuta* DSM 33407. Bile acid resistance profile for (**A**) *Christensenella minuta* DSM 33407 and (**B**) *C. minuta* DSM22607 using OxGall as a source of bile salts. The percentage of growth inhibition was obtained by enumerating the CFU for (**C**) *Christensenella minuta* DSM 33407 and (**D**) *C. minuta* DSM 22607 after 48 h and 96 h of growth in the presence of the corresponding OxGall concentrations. All experiments were performed in triplicates except for the CFU enumerations (C and D; no replicate). Key: OxG: OxGall.

*Cm*DSM22607 was originally described as highly tolerant to bile [7]. Thus, we first evaluated the bile acid resistance potential of the novel strain *Cm*DSM33407 and compared it to the type strain *Cm*DSM22607. For this purpose, the growth of both strains was examined in modified GAM broth medium supplemented or not with a mixture of ox-derived bile acids (i.e., OxGall powder) at increasing concentrations (Figure 1A,B). We observed that both strains were highly resistant to bile acids since they were still able to grow in the presence of 8% of OxGall, the equivalent of 80% of bile. Our results indicated a similar growth to the no-bile control condition in presence of up to 20% of bile. The growth was then slightly reduced at higher concentrations of bile (up to 40% of bile or 4% OxGall) and significantly inhibited when compared to the growth control at higher bile concentrations for both strains (Figure 1A,B).

To determine the proportion of surviving cells in the different bile concentrations after 48 h and 96 h of incubation, 10-fold serial dilutions were plated in order to calculate the Colony-Forming Unit (CFU)/mL for each condition (Figure 1C,D). Overall, we observed a higher sensitivity to bile acids exposure of *Cm*DSM33407 compared with *Cm*DSM22607 that reached 49% and 31% of growth inhibition in presence of 8% OxGall after 48 h for *Cm*DSM33407 and *Cm*DSM22607, respectively. Nevertheless, these results indicate a robust ability to endure bile acid stress at high concentrations. Such resistance mechanisms are typically mediated by the cell membrane constitution and conformation, the presence of efflux pumps and by the action of detoxifying enzymes such as BSH.

### 3.2. Christensenella minuta DSM 33407 and DSM 22607 Carry an Active Bile Salt Hydrolase Enzyme

**Figure 2:**
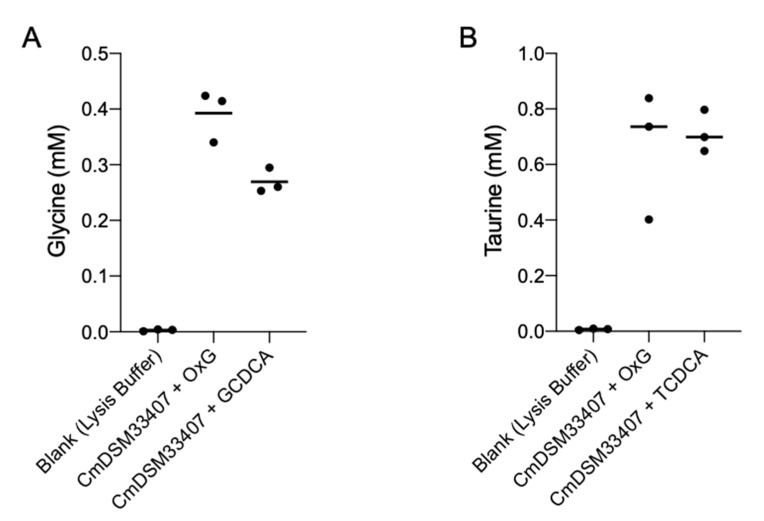
Bile acid deconjugation by *Christensenella minuta* DSM 33407. (**A**) Quantification of glycine (**B**) and taurine release from OxGall, GCDCA and TCDCA by bacteria cellular lysates of *C. minuta* DSM 33407 harvested after 72 h of growth. All experiments were performed in triplicates. Key: OxG: OxGall, GCDCA: glycochenodeoxycholic acid, TCDCA: taurochenodeoxycholic acid.

*Cm*DSM33407 was further selected for the identification of a BSH gene based on its ability to hydrolyze conjugated bile acids in a preliminary activity screening (Figure 2A,B). BSH activity was assayed from bacterial cultures harvested during stationary growth phase. The cell suspensions were lysed and used to measure a putative BSH activity against a mixture of natural bile acids (OxGall), GCDCA and TCDCA. In a typical enzymatic reaction, the release of free glycine and free taurine was measured as an indicator of glycine and taurine hydrolysis from conjugated bile acids by BSH. As displayed in Figure 2A,B, the cell lysate from *Cm*DSM33407 showed a strong ability to release glycine and taurine from all three substrates, indicating that the bacteria expressed an active BSH protein.

**Figure 3:**
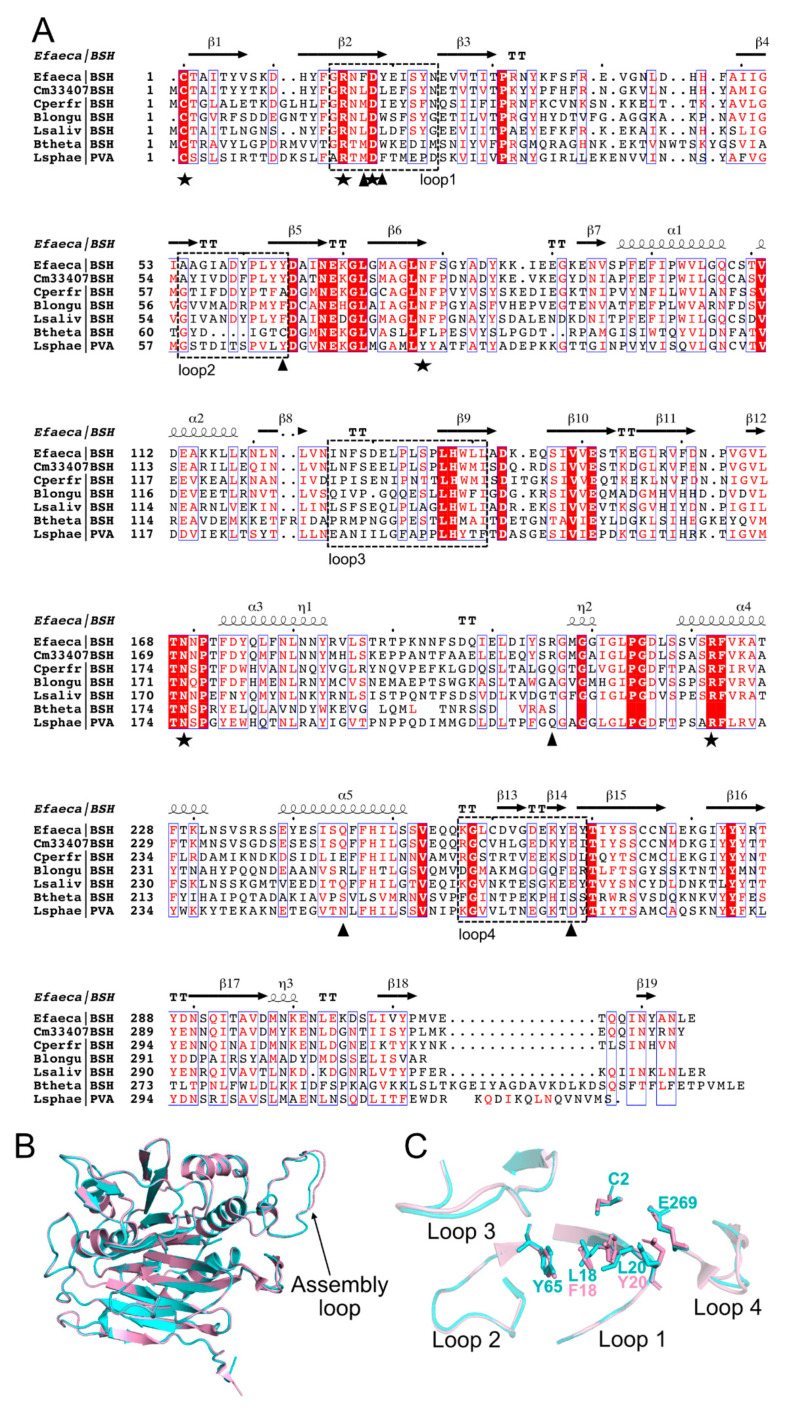
Alignment of the structure-based protein sequence of BSH from *C. minuta* DSM33407 and representative BSH and PVA members. (**A**) Full protein sequence alignment of 325 amino acids numbered. See Table S1 for accession numbers and references. Conserved residues are highlighted in red. Black stars indicate the amino acid residues that are conserved in the choloylglycine hydrolase superfamily. Black triangles indicate the amino acid residues that were shown to be important in catalysis and substrate binding in previous studies [43]. The dashed black rectangles indicate the four substrate binding pocket loops (also represented in panel (B)). (**B**) Tertiary structure homology model of *Cm*33407BSH. Superposition of a BSH Phyre2 homology model [33] (cyan) with the BSH from *Enterococcus faecalis* str. T2 (*Ef*BSH; PDB ID 4WL3 [19], light pink). (**C**) Superposed image of the four substrate binding pocket loops and comparison of *Cm*33407BSH and *Ef*BSH. The image shows the conservation of the key residues within the four loops, including Cysteine (“C”), Tyrosine (“Y”) and Glutamic acid (“E”) in positions 2, 65 and 269, respectively, with the notable exception of the Phenylalanine (“F”) and Tyrosine (“Y”) (light pink) for *Ef*BSH and Leucines (“L”) (cyan) for *Cm*33407BSH residues located at position 18 and 20 respectively. Key: Efaeca: *Enterococcus faecalis* T2; Cm33407: *C. minuta* DSM33407; Cperfr: *Clostridium perfringens*; Blongu: *Bifidobacterium longum*; Lsaliv: *Lactobacillus salivarius*; Btheta: *Bacteroides thetaiotaomicron*; Lsphae: *Lysinibacillus sphaericus*.

Hence, to rapidly identify putative BSH genes, the genome sequences of *Cm*DSM33407 and *Cm*DSM22607 were screened for linear amide C-N hydrolase proteins that encompass choloylglycine hydrolase (conjugated bile acid hydrolase, CBAH, EC:3.5.1.24), penicillin acylase (EC:3.5.1.11) and acid ceramidase (EC:3.5.1.23). Our bioinformatic analysis determined that both *Christensenella minuta* strains included in this study encoded one putative BSH, *Cm33407BSH* (GenBank: MZ028606) and *Cm22607BSH* (GenBank: AYH40638.1). Both protein sequences are strictly identical and the *Cm33407BSH* sequence contained 325 amino acid residues (Figure S2). Sequence alignment with structurally defined BSH and PVA (Penicillin V acylase) sequences exhibited 67.9%, 45.37%, 38.14%, 54.63%, 20.47% and 31.48% identity with *Enterococcus faecalis* T2 (BSH), *Clostridium perfringens* (BSH), *Bifidobacterium longum* (BSH), *Lactobacillus salivarius* (BSH), *Bacteroides thetaiotaomicron* VPI-5482 (BSH) and *Lysinibacillus sphaericus* (PVA), respectively (Figure 3).

The amino acid sequence alignment revealed conservation of the secondary structure organization and of the key active-site residues Cys2, Arg16, Asp19, Asn79, Asn170 and Arg223 (numbering is done according to *Cm*33407BSH sequence; highlighted as black stars in the multiple sequence alignment) as previously described within the large choloylglycine hydrolase family (CGH) [43] (Figure 3A). The Cys2 acts as the catalytic nucleophilic residue. An interesting difference is the Asn79, which is usually replaced by an aromatic amino acid (Phe, Tyr, Trp) within the PVA members as exemplified by the sequence from *Lysinibacillus sphaericus* included in our analysis. Moreover, residues within four loops were conservatively altered (Figure 3A; dashed rectangle): 15–25 (loop 1), 55–65 (loop 2), 127–142 (loop 3), 258–270 (loop 4). These four loops constitute the substrate binding pocket archetype of the CGH family. Notable amino acid differences are Leu18, Leu20, Y65 and E269 residues located within loops 1, 2 and 4, which were only observed in *Cm*33407BSH. These residues were shown to be important in the substrate binding step [44].

*Cm*33407BSH shared 68% sequence identity with an *Enterococcus faecalis* BSH (EfBSH; PDB code 4WL3 [19]) and, as such, was amenable to tertiary structure homology modeling. Phyre2 [33], a protein structure prediction software, utilized PDB code 4WL3 as the sole template, and 321 out of 325 residues (99% of the sequence) were modeled with 100% confidence (Figure 3B). Overall, the model suggests that *Cm*33407BSH presents two antiparallel β-sheets sandwiched between α-helices and adopting the core catalytic four-layered αββα fold of the *N*-terminal nucleophile (Ntn)-hydrolases superfamily. The superimposed structure of *Cm*33407BSH with *Ef*BSH revealed similarities among their structures and their catalytic active sites contain the nucleophile residue (Cys2) (Figure 3C). Notably, four loops constituting the substrate binding pocket were modeled, as well as an assembly loop that holds the protein multimer. All key residues for binding and catalysis are conserved between the two proteins, preserving the binding pocket conformation, with the striking difference of Leu 18 and Leu 20. Indeed, the two aromatic and hydrophobic residues, F18 and Y20, in *Ef*BSH are replaced by Leu residues, a more hydrophobic amino acid without an aromatic ring.

### 3.3. Cm33407BSH Carries a Potent BSH Activity in C. minuta DSM33407

**Figure 4:**
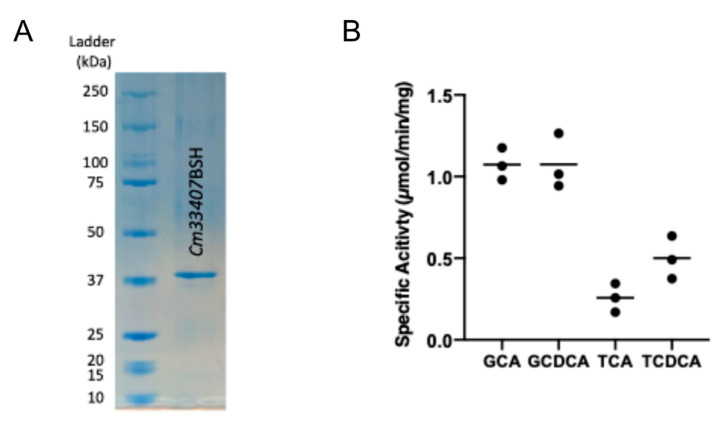
Specific BSH activity of purified *Cm*33407BSH. (**A**) SDS–PAGE of recombinant *Cm*33407BSH protein (the calculated molecular weight of the recombinant *Cm*33407BSH is 39.97 kDa). (**B**) Specific activity of purified BSH from *C. minuta* DSM33407 on four major primary conjugated bile acids. All experiments were performed in triplicate. Key: GCA: glycocholic acid, GCDCA: glycochenodeoxycholic acid, TCA: taurocholic acid, TCDCA: taurochenodeoxycholic acid.

*Cm33407*BSH was produced recombinantly in *Escherichia coli* to investigate the ability of the putative bile salt hydrolase to remove the conjugated amino acid from the steroid core. Neither type I/II signal peptides nor *N*-terminal Cys lipidation were predicted in the signal peptide analysis conducted with SignalP, LipoP and PSORTb, indicating an intracellular localization of the enzyme [28,29,30]. The expression vector pMCSG53 harboring the *Cm33407*BSH-encoding gene was transformed into *E. coli* BL21 (DE3) to overexpress the protein. After IPTG induction and purification with nickel affinity chromatography, a recombinant *Cm33407*BSH single protein band was observed on SDS–PAGE around 40 kDa, which corresponds to the calculated molecular mass of the recombinant *Cm33407*BSH protein (39,966.71 Da) (Figure 4A).

To determine the hydrolytic activity and the substrate specificity of *Cm*33407BSH, the recombinant protein was screened against four major glycine- or taurine-conjugated bile salts. We anticipated that *Cm*33407BSH would be maximally active on glycine-conjugated bile acids based on its family membership. Thus, we used the GCA substrate to determine the optimal pH of the recombinant protein. A bell-shaped pH rate profile was obtained, with the highest enzymatic activity observed at pH 5.0 in 50 mM citrate buffer (Figure S2). In addition, the recombinant protein *Cm*33407BSH displayed high specific activity values for the glycine conjugates GCA and GCDCA (1.073 ± 0.1 μmol·min^−1^·mg^−1^ and 1.074 ± 0.17 μmol·min^−1^·mg^−1^, respectively) (Figure 4B), whereas its specific activity for taurine conjugates was approximately 4.2- and 2.2-fold lower for TCA (0.26 ± 0.09 μmol·min^−1^·mg^−1^) and TCDCA (0.5 ± 0.13 μmol·min^−1^·mg^−1^) at the highest tested substrate concentration (5mM). Together, this indicates that *Cm*33407BSH has a strong preference for glycine-conjugated bile acids as natural substrates.

### 3.4. The BSH Protein Carried by C. minuta sp. Clusters away from all Identified Putative BSH Sequences in the Human Gut Microbiome

**Figure 5:**
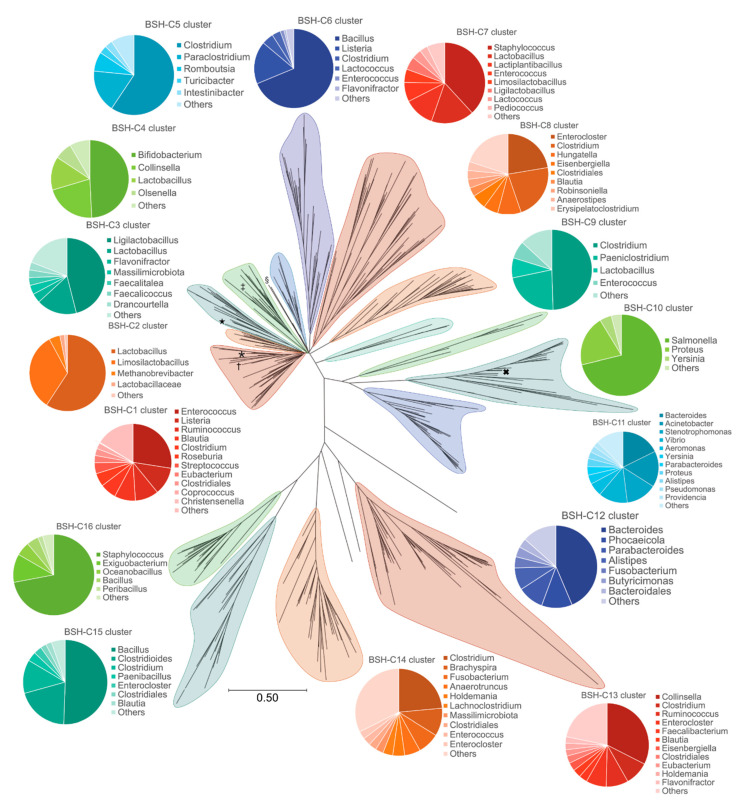
Phylogenetic tree of BSHs from the human gut. A dataset of 6701 BSH sequences from the human gut was selected and processed as described in the Materials and Methods section. Pie charts around the tree show the count of sequences belonging to the mentioned genera for each BSH cluster (see Table S6 for raw data). The asterisk indicates the approximate position of the Christensenellaceae branch. The symbols show the approximate position of branches where characterized BSH can be found (†: WP_002355428.1 with PDB structure 4WL3; §: WP_003461725.1 with PDB structure 2BJF; ‡: WP_013410903.1 with PDB structure 2HF0; ★: WP_047036229.1; with PDB structure 5HKE; ✕: WP_008761025.1 with PDB structure 6UFY).

**Figure 6:**
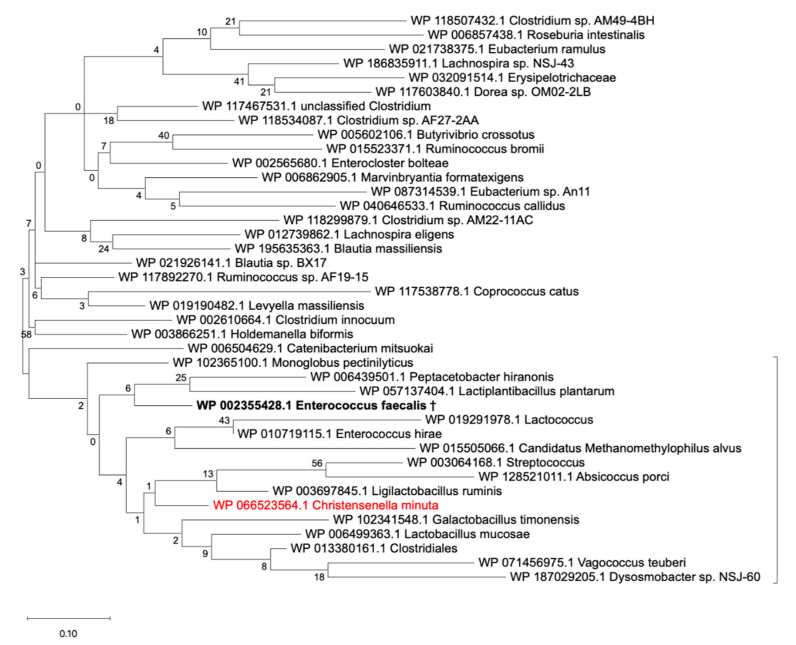
Phylogenetic tree of the BSH-C1 cluster. Bootstrap values are shown on each branch. The branch of the cluster containing the characterized BSH from *Enterococcus faecalis* is in bold (†: WP_002355428.1 with PDB structure 4WL3). The branch containing the *C. minuta*’s BSH sequence is colored in red. The sequences from the clusters within the bracket were extracted and processed as mentioned in the Materials and Methods section to produce the phylogenetic tree shown in Figure S4.

We then decided to interrogate how specific *Cm*33407BSH is within known human metagenomes and therefore examined the distribution of BSH amongst the constituent members of the human gut microbiota. To investigate the sequence diversity within the BSH family, we first collected a non-redundant set of all available reference BSH sequences that could be associated with this family from public databases. As described in the flowchart in Figure S1, we first extracted all protein sequences annotated as choloylglycine hydrolase, bile salt hydrolase, linear amide C-N hydrolase and choloylglycine hydrolase family protein from the NCBI protein database. This step allowed us to retrieve a total of 14,319 sequences putatively annotated as BSH enzymes (Table S2). To refine this dataset by only selecting the sequences that can be found in the human gut, we blasted all 14,319 sequences against the gastrointestinal tract Human Microbiome Project (HMP) protein database and against the Unified Human Gastrointestinal Protein (UHGP) database [36,38,39]. Here, we obtained drastically different results since the HMP database only recovered 1658 sequences at 100% identity with a BSH annotation while the UHGP blast returned 5179 sequences at this threshold (Figure S3, Table S3). We thus decided to continue the analysis using the UHGP annotated sequences as a representative catalog of the proteins carried by the human microbiota. Using a minimum threshold of identity of 85%, 6701 sequences out of 14,319 were retrieved as putative BSH proteins from the human gut (see Figure S1 and Table S4 for a list of raw data). These sequences were all associated with an E-value of 0 and the lowest coverage was 86%, which gives high confidence in the overall quality of the dataset. In order to construct a phylogenetic tree of the putative BSH sequences from the human gut microbiome, we clustered these 6701 sequences into 413 clusters of sequences using a 70% identity threshold (see Table S5 for raw data). The reference sequences of the 413 clusters were used to perform a phylogenetic analysis: the sequences were aligned, and the phylogenetic tree obtained is presented in Figure 5. Based on the topology of the tree, 16 major clusters were identified (see Table S6 for raw data). The christensenella subcluster was thus identified within the BSH-C1 cluster (Figure 5) largely dominated by *Enterococcus* spp. sequences. A detailed analysis of the C1 cluster demonstrated that *C. minuta’s* BSH sequence was isolated on a separate branch from any other bacterial group (Figure 6). Since each branch from the tree represents a cluster of sequences sharing 70% identity or more, each branch isolates sequences that have less than 70% identity against any other branch. Zooming again on the sub-cluster indicated by a bracket on Figure 6, we identified that *C. minuta’s* BSH sequences are located within a specific cluster of *Christensenellaceae* sequences (see Figure S5 and Table S7 for raw data). Thus, the *C. minuta’s* BSH sequences are clearly different from the rest of the putative BSH protein sequences found in the gut microbiota.

## 4. Discussion

The objective of this work was to explore the heterologous expression of an active BSH enzyme by the *Christensenellaceae* species *Christensenella minuta*. We determined that indeed this species encodes for a full and conserved BSH sequence of 325 amino acids. Even if the enzyme was able to hydrolyze both taurine- and glycine-conjugated bile acids, we demonstrated that it had a higher activity for glycine-conjugated bile acids. We did not observe any difference in activity between steroidal core structures (i.e., cholic acid versus chenodeoxycholic acid). Yet, we only tested the human primary bile acids in this work and did not explore other steroid conformations that naturally occur in the gut following bacterial metabolism, neither did we test activity against one of the murine primary bile acids *beta*-muricholic acid.

The BSH family is largely represented in the major bacterial phyla (Firmicutes, Bacteroidetes, Actinobacteria) and even across domains of life (bacteria and archaea) within the gut ecosystem [20,43]. This ubiquitous representation reflects the importance of the protein family in bacterial physiology, which plays an essential role in bile acid resistance [45]. As a consequence, BSH enzymes have been largely studied in pathogens and in potentially beneficial bacteria, and mostly in Gram-positive bacteria [13,17,46,47] Conversely, only a limited number of Gram-negative BSH have been evaluated, of which BSH from *Bacteroides* species [21]. Interestingly, selective BSH enzymes from the gut dwelling community of Gram-negative bacteria have been reported for tauro-conjugated bile acids [21] but not for glycine, while we describe here a new BSH protein with glycine specificity. Considering the relative specificity for glycine-conjugated bile acids, this indicates that *C. minuta* are well adapted to colonize the human gut since humans preferentially make glycine conjugates while rodents, and particularly mice, preferentially produce tauro-conjugated bile acids [48].

In this study, we determined that the optimum activity of *Cm*33407BSH occurs at a pH of 5.0, which is consistent with previously characterized BSHs (optimum pHs ranging from 3.8 to 7.0) and the pH profile in the human large intestine [43,49,50]. Interestingly, the toxicity of glyco-conjugated bile salts has been found to be significantly higher than tauro-conjugated forms, especially at low pHs [51,52]. However, the relative toxicity of conjugated and deconjugated bile acids for specific bacterial strains is still unclear. It is well known that microbial bile acid resistance contributes to a successful colonization of the GI tract environment [48]. Moreover, a recent study conducted in *Lactobacillus* spp. elegantly demonstrated the link between BSH substrate specificity and bacterial fitness to effectively colonize its host [17]. Taken together, our data therefore indicate that *Cm33407BSH* may contribute to the high bile acid resistance observed for *Cm*DSM33407 and that it may play a bile detoxification role conferring an evolutionary advantage to colonize its hosts [20,53,54].

Three-dimensional information is key to provide insights into substrate specificity and catalysis. For example, loops are critical to determine the substrate binding and specificity as they will dictate the volume of the binding site [43,55]. Hence, subtle changes in the loop structures can significantly impact the catalytic activity of the BSH. A limitation of our study is that we did not attempt to obtain crystals of *Cm*33407BSH for experimental tertiary structure determination. However, a few tertiary structures of BSHs are publicly available [16,19,56,57,58]. Thus, we provide here a tertiary structural model built from the closest relative BSH model available and show that only a few key amino acid residues are modified in the portions involved in the structure of the binding pocket. F18, Y20 and Y65 have been proposed to align the substrate in an orientation leading to a higher activity for *Ef*BSH, and both C2 and Y20 act as gatekeeper [19]. Since F18 and Y20 have both been replaced by more hydrophobic leucine residues in *Cm33407BSH*, and despite their relative proximity in the phylogenetic tree, it is likely that the enzyme efficacy is different from the *Enterococcus faecalis* model. Similarly, we observed a modification of Y65 in *L. salivarius* (PDB ID 5HKE), which has been described as involved in the specific activity of the enzyme towards glycine-conjugated bile acids [55,56]. Yet, more studies must be performed to determine this accurately. Nevertheless, these observations strongly support the distinctive nature of *C. minuta*’s BSH.

We finally explored the distribution of putative BSH sequences in the gut microbiome in order to evaluate the sequence of *Cm33407BSH* with regard to the diversity of the gut ecosystem. To perform this analysis, we needed to collect a sequence catalog of all publicly available putative BSH sequences. A similar type of collection was recently published by Song et al. [22] but because *C. minuta*’s BSH gene is publicly annotated as a linear amide *C-N* hydrolase, these sequences were missed. Thus, we adjusted our methods accordingly and included over 9000 additional sequences in our collection from the NCBI refseq protein database (Figure S1). By doing so, we performed the most comprehensive analysis of putative BSH sequences in gut microbiomes to date. In addition, the study by Song et al. only used the HMP catalog, while our analysis showed that using the UHGP database allowed a more accurate identification of the putative BSH proteins from the human gut microbiome. As mentioned by Almeida et al. [36], the UHGP database was built using reference genomes from cultured gut microbiota and Metagenome Assembled Genomes (MAGs) from several studies. Thus, the UHGP database was built from more than 200,000 non-redundant genomes from around 4650 prokaryotes whereas the HMP gastrointestinal tract protein database only contains 457 unique genomes. This additional diversity in the UHGP database allowed an increased specificity in the identification of putative BSH sequences from the human gut microbiome. Interestingly, the number of sequences as well as the number of genera represented in the 16 major BSH clusters identified by the phylogenetic analysis, are uneven between the clusters without any obvious correlation between the two parameters (Figure S6). We hypothesize that this is the result of a bias caused by an overabundance of studies on a few genera such as *Enterococcus* spp., *Staphylococcus* spp., *Bacillus* spp., *Salmonella* spp., *Bacteroides* spp. and *Lactobacillus* spp. [18,19,21]. Nevertheless, although *C. minuta*’s BSH is included in one of the most diverse clusters, both in number of sequences and in number of genera (Figure S6), the phylogenetic analysis revealed that the amino acid sequence of this BSH was unique. Similarly, the characterization of the structure of BSH enzymes has mainly been focused on highly studied genera (available structures in Table S1). Together, these observations indicate that more studies are needed to investigate rare species from the gut microbiome such as *C. minuta* to gain more insights into the diversity of BSH enzymes in the human gut.

Bile salt hydrolysis is the first step of bile acid metabolism. Most unconjugated bile salts are then either passively re-absorbed through the gut epithelium or actively reabsorbed in the terminal ileum. A mere 5% of the bile acid pool travels to the colon where it will undergo further metabolism to result in secondary bile acid production [59]. Unconjugated bile salts are highly toxic for bacteria and thus they act as important regulators of the microbial community [60]. Hence, modulating BSH activity is an effective way to impact the composition of the gut ecosystem. Conversely, a damaged BSH activity has been reported in the chronic inflammatory bowel disorder Crohn’s disease [61,62], which even led to the recent development of a non-invasive diagnostic tool to assess BSH activity in vivo [63]. Another therapeutic implication was recently demonstrated for recurrent *Clostridioides difficile* infections that are effectively treated by fecal microbial transplantation (FMT). In an elegant study, Mullish et al. revealed that FMT restored BSH activity in patients and that this was sufficient to clear the infection in vitro [64]. Hence, there is increasing interest in developing novel therapeutic approaches that aim at repairing BSH activity in the human gut. In the context of metabolic disorders, a recent study reported that non-obese liver fibrotic individuals displayed a significantly lower copy number of BSH encoding genes in their gut microbiome [65]. It has been shown in vivo in animal models that increasing BSH activity in the gut ecosystem led to lower body weight gain, lower adiposity and reduction of both circulating Low Density Lipoprotein (LDL) cholesterol and triglycerides [12]. This study also suggested that enriching gut microbiomes with bacterial strains carrying a highly active BSH may be a strategy to alleviate excess body weight. Given that *Cm*DSM33407 presents strong anti-obesity effects in animal models [8], its BSH activity may be an important keystone function in its mechanism of action that warrants further studies.

## 5. Conclusions

In conclusion, we functionally dissected a novel BSH enzyme, thereby resolving a key outstanding deficit in our understanding of bile acid metabolism by the keystone gut bacterium *C. minuta*. This function may participate in the key role played by this species in healthy gut ecosystems, which warrants further studies to fully understand how *C. minuta* benefits its host through modulations of bile acid metabolism.

## 6. Patents

Part of this work contributes to the data package for a pending patent (FR2012821). Dr. Déjean, Ms. Tudela, Dr. Rawadi and Dr. Claus are part of the inventors list.

## Data Availability

The BSH amino acid sequence from *Christensenella minuta* DSM33407 presented in this study is openly available in the GenBank database (accession: MZ028606).

## Supplementary Materials

The following are available online at https://www.mdpi.com/article/10.3390/microorganisms9061252/s1, Figure S1: Flowchart applied to select BSH sequences, Figure S2: Alignment of the BSH protein sequences from *C. minuta* strainsDSM22607 and DSM33407, Figure S3: pH-rate of recombinant Xla1_01266 BSH enzyme using glycocholic acid as the substrate, Figure S4: Comparison of the HMP and UHGP database, Figure S5: Phylogenetic subtree of the BSH-C1 cluster shown in Figure 6, Figure S6: Diversity of genus and number of sequences in the 16 major BSH clusters, Table S1: Characterized structures identified in the phylogenetic tree, Table S2: Sequences selected from the NCBI protein database, Table S3: Distribution of the identities between the selected BSH sequences and HMP or UHGP database, Table S4: BSH that shared more than 85% identity with at least one of the sequences in the UHGP database, Table S5: Results of clustering (70% identity) on the selected BSH sequences, Table S6: Genus composition of the clusters identified in the phylogenetic tree, Table S7: Sequences from the clusters close to the BSH from *Christensenella minuta* strains. BSH-DBase.fasta: this file contains all putative BSH sequences used to reconstruct the phylogenetic tree presented in Figure 5.
