# Supplementary material for: Identifying a Novel Bile Salt Hydrolase from the Keystone Gut Bacterium Christensenella minuta
Source: Microorganisms. 2021 Jun 9;9(6):1252. doi: 10.3390/microorganisms9061252 (PMC8228234; doi:10.3390/microorganisms9061252)
Supplement: Supplementary file 1 [file microorganisms-09-01252-s001.zip › microorganisms-1225367-supplementary-english.pdf]

Identifying a novel and evolutionarily unique bile salt hydrolase from the keystone gut bacterium *Christensenella minuta*

Guillaume Déjean [1], Héloïse Tudela [1,2], Lisa Bruno [1], Déborah Kissi [1], Georges Rawadi [1] and Sandrine P. Claus [1,*]

[1] Ysopia Bioscience, 17 Place de la Bourse, 33076 Bordeaux, France; guillaume.dejean@ysopia.bio (G.D.); heloise.tudela@ysopia.bio (H.T.); lisa.bruno@ysopia.bio (L.B.); deborah.kissi@ysopia.bio (D.K.); georges.rawadi@ysopia.bio (G.R.)
[2] ImmunoConcEpT, 146 rue Léo Saignat, 33076 Bordeaux, France
* Correspondence: Sandrine.claus@ysopia.bio;

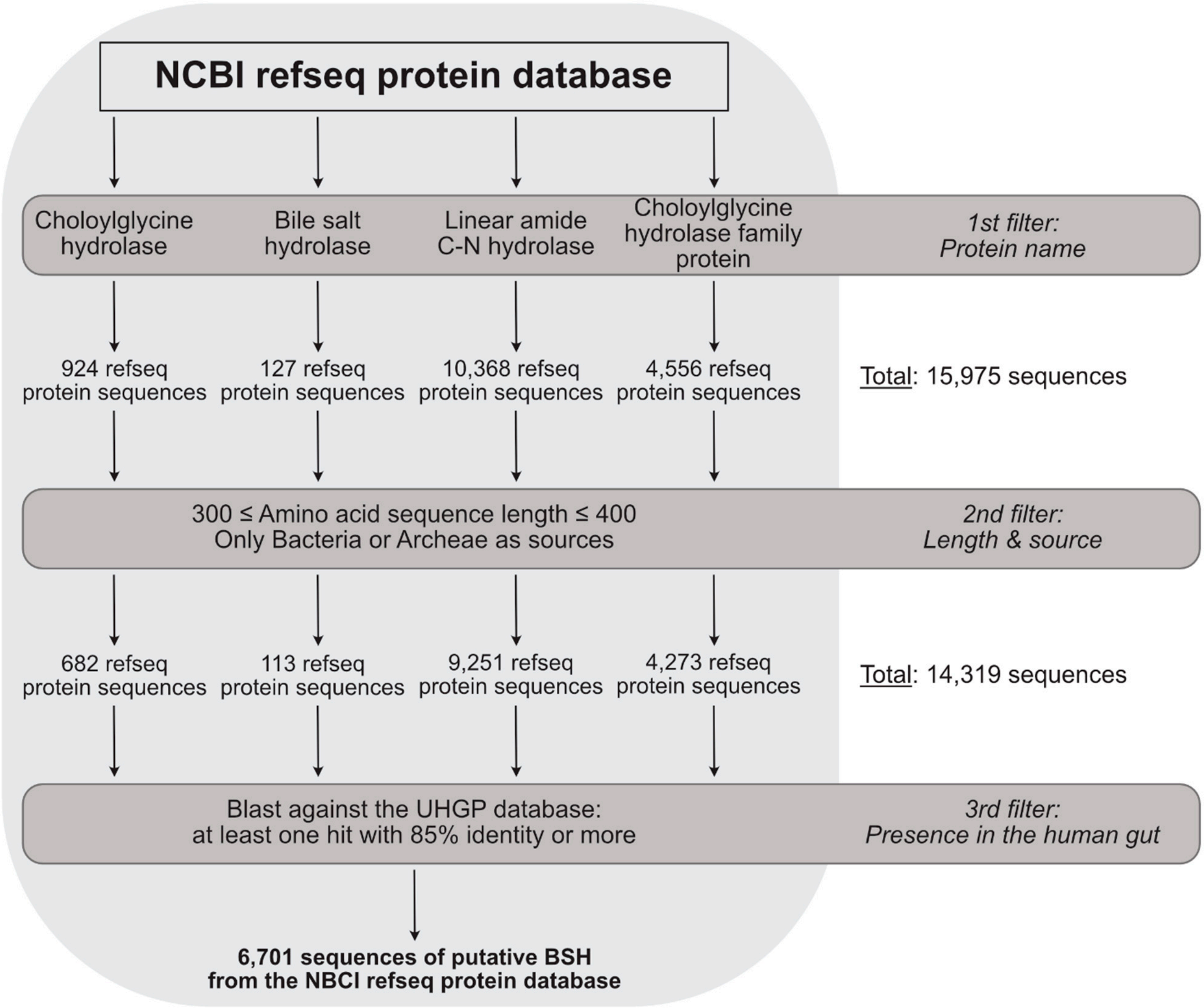

Figure S1. Flowchart applied to select BSH sequences. This flowchart was used to select the BSH sequences included in the phylogenetic and clustering analyses.

```
                        cov    pid   1 [       .         .         .         .         :         .         .         . 80
1 Cm33407BSH         100.0% 100.0%     MCTAITYYTKDHYFGRNLDLEFSYNETVTVTPKYYPFHFRNGKVLNHHYAMIGMAYIVDDFPLYYDATNEKGLSMAGLNF
2 Cm22607BSH         100.0% 100.0%     MCTAITYYTKDHYFGRNLDLEFSYNETVTVTPKYYPFHFRNGKVLNHHYAMIGMAYIVDDFPLYYDATNEKGLSMAGLNF
  consensus/100%                       MCTAITYYTKDHYFGRNLDLEFSYNETVTVTPKYYPFHFRNGKVLNHHYAMIGMAYIVDDFPLYYDATNEKGLSMAGLNF
  consensus/90%                        MCTAITYYTKDHYFGRNLDLEFSYNETVTVTPKYYPFHFRNGKVLNHHYAMIGMAYIVDDFPLYYDATNEKGLSMAGLNF
  consensus/80%                        MCTAITYYTKDHYFGRNLDLEFSYNETVTVTPKYYPFHFRNGKVLNHHYAMIGMAYIVDDFPLYYDATNEKGLSMAGLNF
  consensus/70%                        MCTAITYYTKDHYFGRNLDLEFSYNETVTVTPKYYPFHFRNGKVLNHHYAMIGMAYIVDDFPLYYDATNEKGLSMAGLNF

                        cov    pid  81         .         1         .         .         .         .         :         . 160
1 Cm33407BSH         100.0% 100.0%     PDNADYKEVKEGYDNIAPFEFIPWILGQCASVSEARILLEQINLVNLNFSEELPLSPLHWMISDQRDSIVVESTKDGLKV
2 Cm22607BSH         100.0% 100.0%     PDNADYKEVKEGYDNIAPFEFIPWILGQCASVSEARILLEQINLVNLNFSEELPLSPLHWMISDQRDSIVVESTKDGLKV
  consensus/100%                       PDNADYKEVKEGYDNIAPFEFIPWILGQCASVSEARILLEQINLVNLNFSEELPLSPLHWMISDQRDSIVVESTKDGLKV
  consensus/90%                        PDNADYKEVKEGYDNIAPFEFIPWILGQCASVSEARILLEQINLVNLNFSEELPLSPLHWMISDQRDSIVVESTKDGLKV
  consensus/80%                        PDNADYKEVKEGYDNIAPFEFIPWILGQCASVSEARILLEQINLVNLNFSEELPLSPLHWMISDQRDSIVVESTKDGLKV
  consensus/70%                        PDNADYKEVKEGYDNIAPFEFIPWILGQCASVSEARILLEQINLVNLNFSEELPLSPLHWMISDQRDSIVVESTKDGLKV

                        cov    pid 161         .         .         .         2         .         .         .         . 240
1 Cm33407BSH         100.0% 100.0%     FENPVGVLTNNPTFDYQMFNLNNYMHLSKEPPANTFAAELELEQYSRGMGAIGLPGDLSSASRFVKAAFTKMNSVSGDSE
2 Cm22607BSH         100.0% 100.0%     FENPVGVLTNNPTFDYQMFNLNNYMHLSKEPPANTFAAELELEQYSRGMGAIGLPGDLSSASRFVKAAFTKMNSVSGDSE
  consensus/100%                       FENPVGVLTNNPTFDYQMFNLNNYMHLSKEPPANTFAAELELEQYSRGMGAIGLPGDLSSASRFVKAAFTKMNSVSGDSE
  consensus/90%                        FENPVGVLTNNPTFDYQMFNLNNYMHLSKEPPANTFAAELELEQYSRGMGAIGLPGDLSSASRFVKAAFTKMNSVSGDSE
  consensus/80%                        FENPVGVLTNNPTFDYQMFNLNNYMHLSKEPPANTFAAELELEQYSRGMGAIGLPGDLSSASRFVKAAFTKMNSVSGDSE
  consensus/70%                        FENPVGVLTNNPTFDYQMFNLNNYMHLSKEPPANTFAAELELEQYSRGMGAIGLPGDLSSASRFVKAAFTKMNSVSGDSE

                        cov    pid 241         :         .         .         .         .         3         .         . 320
1 Cm33407BSH         100.0% 100.0%     SESISQFFHILGSVEQQRGCVHLGEDKYEITIYSSCCNMDKGIYYYTTYENNQITAVDMYKENLDGNTIISYPLMKEQQI
2 Cm22607BSH         100.0% 100.0%     SESISQFFHILGSVEQQRGCVHLGEDKYEITIYSSCCNMDKGIYYYTTYENNQITAVDMYKENLDGNTIISYPLMKEQQI
  consensus/100%                       SESISQFFHILGSVEQQRGCVHLGEDKYEITIYSSCCNMDKGIYYYTTYENNQITAVDMYKENLDGNTIISYPLMKEQQI
  consensus/90%                        SESISQFFHILGSVEQQRGCVHLGEDKYEITIYSSCCNMDKGIYYYTTYENNQITAVDMYKENLDGNTIISYPLMKEQQI
  consensus/80%                        SESISQFFHILGSVEQQRGCVHLGEDKYEITIYSSCCNMDKGIYYYTTYENNQITAVDMYKENLDGNTIISYPLMKEQQI
  consensus/70%                        SESISQFFHILGSVEQQRGCVHLGEDKYEITIYSSCCNMDKGIYYYTTYENNQITAVDMYKENLDGNTIISYPLMKEQQI

                        cov    pid 321     ] 325
1 Cm33407BSH         100.0% 100.0%     NYRNY
2 Cm22607BSH         100.0% 100.0%     NYRNY
  consensus/100%                       NYRNY
  consensus/90%                        NYRNY
  consensus/80%                        NYRNY
  consensus/70%                        NYRNY
```

Figure S2. Alignment of the BSH protein sequences from *C. minuta* strains DSM22607 and DSM33407. The amino acid sequence from the BSH of *C. minuta* DSM22607 (*Cm*22607BSH) and *C. minuta* DSM33407 (*Cm*33407BSH) were aligned using Muscle (v.3.8.31) and the alignment was viewed using MView.

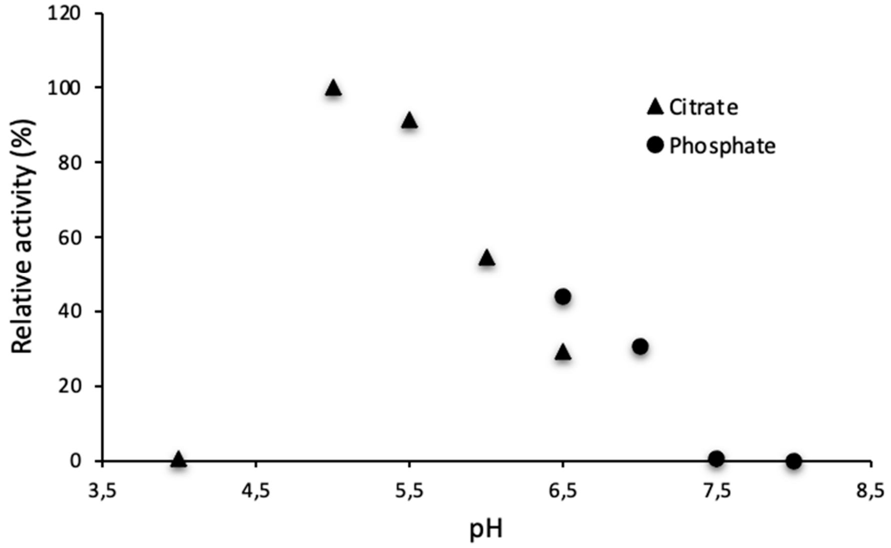

Figure S3. pH-rate profile of recombinant *Cm*33407BSH enzyme using glycocholic acid as the substrate.

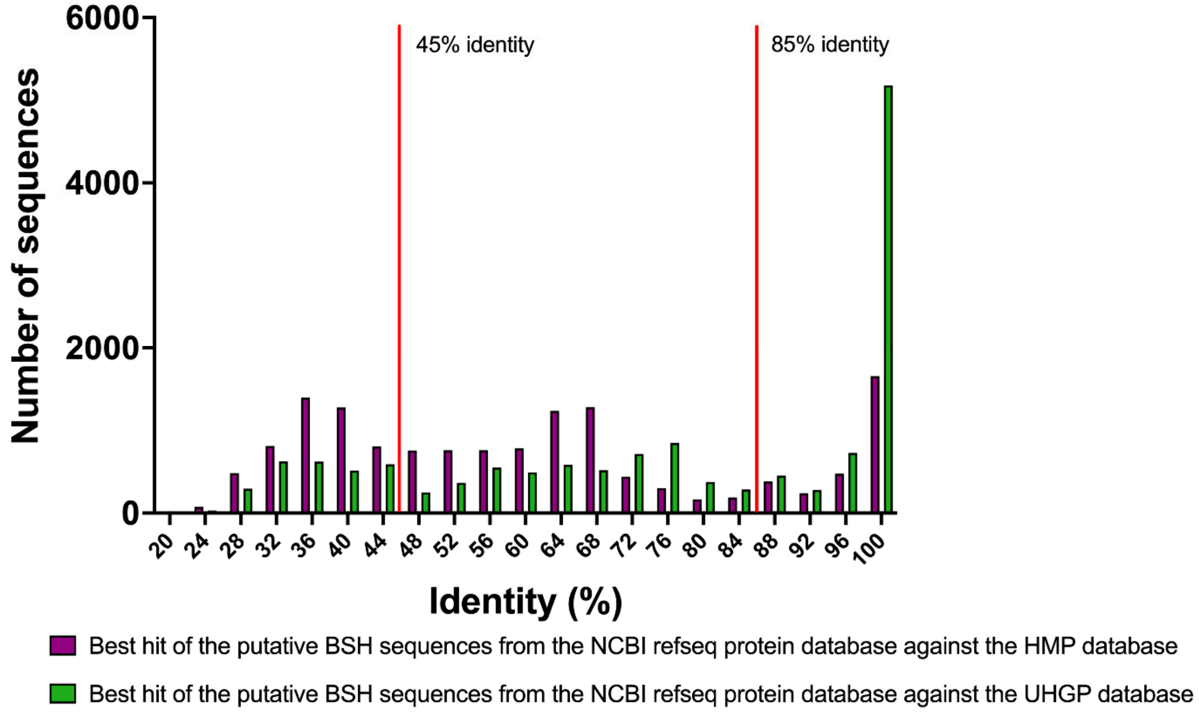

Figure S4. Comparison of the HMP and UHGP databases. The 14,320 sequences extracted from the NCBI refseq database using the flowchart shown in Figure S1 were blasted against the gastrointestinal tract Human Microbiome Project (HMP) protein and the Unified Human Gastrointestinal Protein (UHGP) databases.

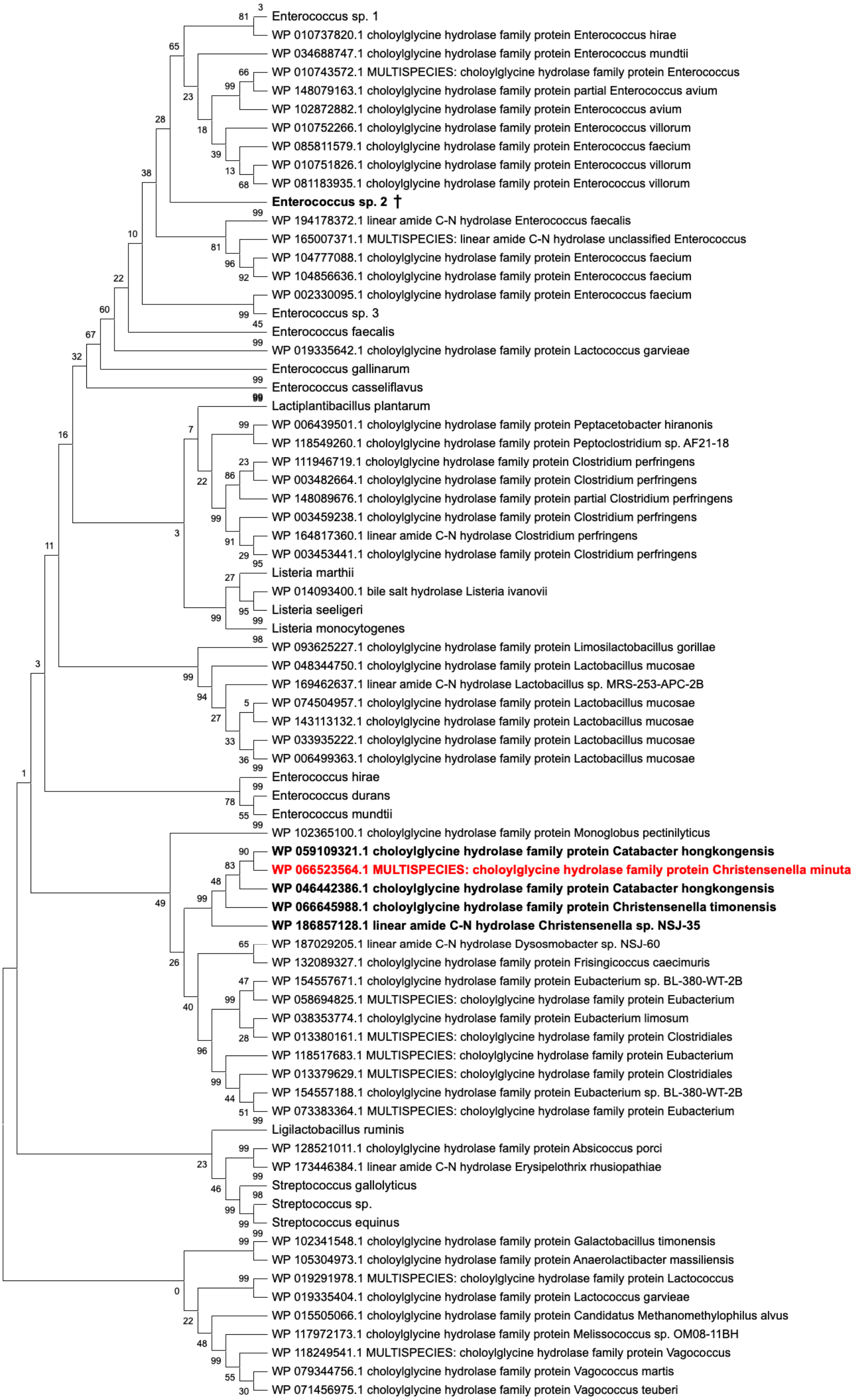
Enterococcus sp. 1
WP 010737820.1 choloylglycine hydrolase family protein Enterococcus hirae
WP 034688747.1 choloylglycine hydrolase family protein Enterococcus mundtii
WP 010743572.1 MULTISPECIES: choloylglycine hydrolase family protein Enterococcus
WP 148079163.1 choloylglycine hydrolase family protein partial Enterococcus avium
WP 102872882.1 choloylglycine hydrolase family protein Enterococcus avium
WP 010752266.1 choloylglycine hydrolase family protein Enterococcus villorum
WP 085811579.1 choloylglycine hydrolase family protein Enterococcus faecium
WP 010751826.1 choloylglycine hydrolase family protein Enterococcus villorum
WP 081183935.1 choloylglycine hydrolase family protein Enterococcus villorum
Enterococcus sp. 2 †
WP 194178372.1 linear amide C-N hydrolase Enterococcus faecalis
WP 165007371.1 MULTISPECIES: linear amide C-N hydrolase unclassified Enterococcus
WP 104777088.1 choloylglycine hydrolase family protein Enterococcus faecium
WP 104856636.1 choloylglycine hydrolase family protein Enterococcus faecium
WP 002330095.1 choloylglycine hydrolase family protein Enterococcus faecium
Enterococcus sp. 3
Enterococcus faecalis
WP 019335642.1 choloylglycine hydrolase family protein Lactococcus garvieae
Enterococcus gallinarum
Enterococcus casseliflavus
Lactiplantibacillus plantarum
WP 006439501.1 choloylglycine hydrolase family protein Peptacetobacter hiranonis
WP 118549260.1 choloylglycine hydrolase family protein Peptoclostridium sp. AF21-18
WP 111946719.1 choloylglycine hydrolase family protein Clostridium perfringens
WP 003482664.1 choloylglycine hydrolase family protein Clostridium perfringens
WP 148089676.1 choloylglycine hydrolase family protein partial Clostridium perfringens
WP 003459238.1 choloylglycine hydrolase family protein Clostridium perfringens
WP 164817360.1 linear amide C-N hydrolase Clostridium perfringens
WP 003453441.1 choloylglycine hydrolase family protein Clostridium perfringens
Listeria marthii
WP 014093400.1 bile salt hydrolase Listeria ivanovii
Listeria seeligeri
Listeria monocytogenes
WP 093625227.1 choloylglycine hydrolase family protein Limosilactobacillus gorillae
WP 048344750.1 choloylglycine hydrolase family protein Lactobacillus mucosae
WP 169462637.1 linear amide C-N hydrolase Lactobacillus sp. MRS-253-APC-2B
WP 074504957.1 choloylglycine hydrolase family protein Lactobacillus mucosae
WP 143113132.1 choloylglycine hydrolase family protein Lactobacillus mucosae
WP 033935222.1 choloylglycine hydrolase family protein Lactobacillus mucosae
WP 006499363.1 choloylglycine hydrolase family protein Lactobacillus mucosae
Enterococcus hirae
Enterococcus durans
Enterococcus mundtii
WP 102365100.1 choloylglycine hydrolase family protein Monoglobus pectinilyticus
WP 059109321.1 choloylglycine hydrolase family protein Catabacter hongkongensis
WP 066523564.1 MULTISPECIES: choloylglycine hydrolase family protein Christensenella minuta
WP 046442386.1 choloylglycine hydrolase family protein Catabacter hongkongensis
WP 066645988.1 choloylglycine hydrolase family protein Christensenella timonensis
WP 186857128.1 linear amide C-N hydrolase Christensenella sp. NSJ-35
WP 187029205.1 linear amide C-N hydrolase Dysosmobacter sp. NSJ-60
WP 132089327.1 choloylglycine hydrolase family protein Frisingicoccus caecimuris
WP 154557671.1 choloylglycine hydrolase family protein Eubacterium sp. BL-380-WT-2B
WP 058694825.1 MULTISPECIES: choloylglycine hydrolase family protein Eubacterium
WP 038353774.1 choloylglycine hydrolase family protein Eubacterium limosum
WP 013380161.1 MULTISPECIES: choloylglycine hydrolase family protein Clostridiales
WP 118517683.1 MULTISPECIES: choloylglycine hydrolase family protein Eubacterium
WP 013379629.1 MULTISPECIES: choloylglycine hydrolase family protein Clostridiales
WP 154557188.1 choloylglycine hydrolase family protein Eubacterium sp. BL-380-WT-2B
WP 073383364.1 MULTISPECIES: choloylglycine hydrolase family protein Eubacterium
Ligilactobacillus ruminis
WP 128521011.1 choloylglycine hydrolase family protein Absicoccus porci
WP 173446384.1 linear amide C-N hydrolase Erysipelothrix rhusiopathiae
Streptococcus gallolyticus
Streptococcus sp.
Streptococcus equinus
WP 102341548.1 choloylglycine hydrolase family protein Galactobacillus timonensis
WP 105304973.1 choloylglycine hydrolase family protein Anaerolactibacter massiliensis
WP 019291978.1 MULTISPECIES: choloylglycine hydrolase family protein Lactococcus
WP 019335404.1 choloylglycine hydrolase family protein Lactococcus garvieae
WP 015505066.1 choloylglycine hydrolase family protein Candidatus Methanomethylophilus alvus
WP 117972173.1 choloylglycine hydrolase family protein Melissococcus sp. OM08-11BH
WP 118249541.1 MULTISPECIES: choloylglycine hydrolase family protein Vagococcus
WP 079344756.1 choloylglycine hydrolase family protein Vagococcus martis
WP 071456975.1 choloylglycine hydrolase family protein Vagococcus teuberi

Figure S5. Phylogenetic subtree of the BSH-C1 cluster shown in Figure 6. Bootstrap values are shown on the branches. The composition of collapsed branches is described in the Supplementary Materials. The branch of the cluster containing the characterized BSH from *Enterococcus faecalis* is displayed in bold († : WP_002355428.1 with PDB structure 4WL3). The BSH from *C. minuta* is shown in red; the BSHs from other *Christensenellaceae* and *Catabacteriaceae* are shown in bold.

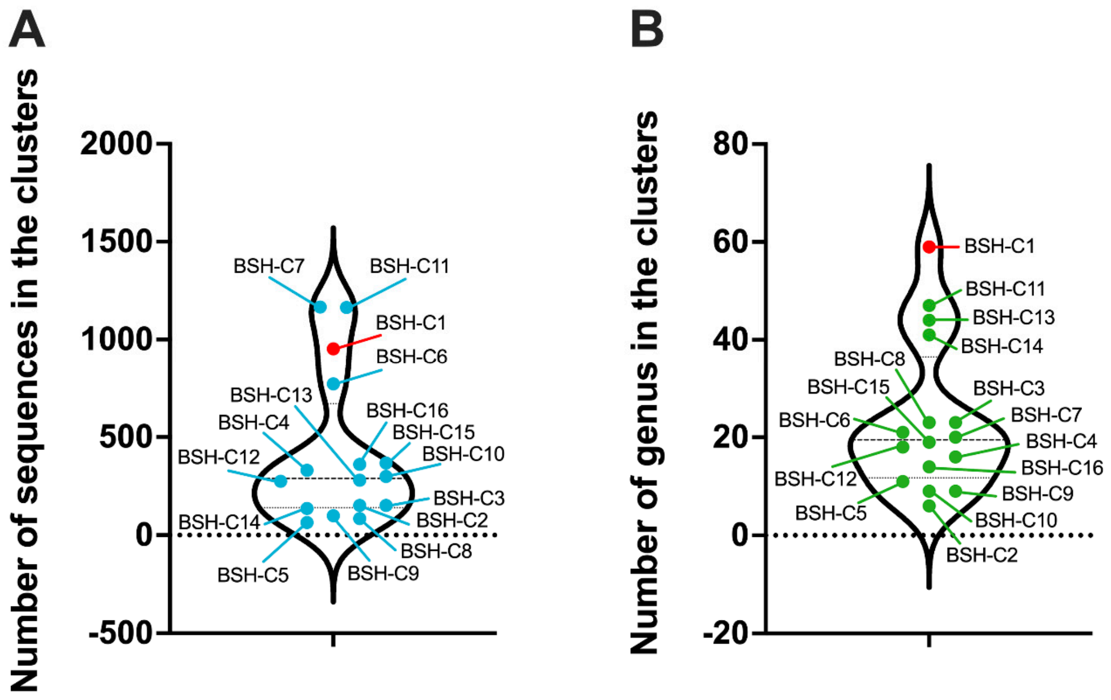

Figure S6. Diversity of genus and number of sequences in the 16 major BSH clusters identified in Figure 4. Each dot corresponds to the number of BSH sequences (panel **A**) or the number of genera (panel **B**) contained in each major cluster from Figure 4. The BSH-C1 cluster is shown in red in both panels. The violin plots were generated using GraphPad Prism v9.1.0.
